# Honey Antibacterial Effect Boosting Using* Origanum vulgare L*. Essential Oil

**DOI:** 10.1155/2018/7842583

**Published:** 2018-03-15

**Authors:** Hamada Imtara, Youssef Elamine, Badiâa Lyoussi

**Affiliations:** Laboratory of Physiology, Pharmacology and Environmental Health, Faculty of Sciences Dhar El Mehraz, University Sidi Mohamed Ben Abdallah, BP 1796 Atlas, 30 000 Fez, Morocco

## Abstract

The appearance of new bacterial strains which cause pathogenic diseases and which are resistant to the most used antibiotics requires probing new antibacterial agents sources. Therefore, the main aim of the present work was to follow the antibacterial activity of honey samples from Palestine and Morocco, after the combination with* Origanum vulgare L.* essential oil, and figure out whether the honey physicochemical parameters and geographic origin influence the final activity. The results of this study showed good geographical discrimination between the Palestinians and Moroccan honey samples. The antioxidant and antimicrobial activities showed a significant correlation with honey color, melanoidins, and phenolic and flavonoids contents. Furthermore, the possible effect of honey physicochemical parameters on the gained antimicrobial activities was assessed using the principal component analysis (PCA). Some parameters showed a promising effect and seem to be important in the process of honey samples selection. Namely, melanoidins content, phenolic content, electrical conductivity, and mineral content were shown to be positively influencing the gained antibacterial activity after the combination with essential oil against the tested strains, although a significant negative correlation was seen with the FIC only in the case of* Escherichia coli* (ATB: 57).

## 1. Introduction

Multidrug resistant bacteria or superbugs pose a serious threat to the world health [1]. The world health organization published in the beginning of the current year (2017) a report listing the most dangerous superbugs to which new antibiotics should be discovered urgently [2]. The decrease of introduction of new antibiotics to the market contributed to the emergence and spreading of superbugs to an uncontrollable extent [3]. The discovery of new antibacterial agents is mainly based on natural compounds such as nonculturable bacteria as targets and nonnatural chemical route, for example, prontosil, metronidazole, and isoniazid [4]. Another source is the screening of the bioactive compounds provided by the natural products [5].

Since ancient times, honey was used for burns and wounds healing [6, 7]. Subjecting honey to laboratory and clinical investigations during the past few decades linked its healing and anti-inflammatory properties [8] to the antimicrobial effect but also antioxidant activity [9, 10]. Honey antimicrobial property, as the main factor in the protection of the wound, is mainly due to the osmolarity effect of high sugar concentrations, low water content, and low pH and content of other compounds [11–13]. In addition, honey contains molecules that inhibit bacterial growth, such as hydrogen peroxide and nonperoxide inhibins, as well-known as phytochemicals compounds [14, 15]. The antimicrobial activity of honey seems to vary depending on its geographical and/or botanical origins [16]. The antimicrobial specificity of honey also depends on the tested pathogen [7, 17].

Essential oils possess antiradical, antioxidant, antibacterial, antiparasitic, antifungal, and antiviral properties [18, 19]. An interesting essential oil that has been recognized as source of alternative antimicrobial and antioxidant compounds to be applied in food conservation is extracted from* Origanum vulgare L.* (Lamiaceae). It is a very versatile plant and has been used in traditional health care for a long time such as carminative, antispasmodic, and antiseptic. Further, its biological properties have been explored by pharmaceutical, culinary, agricultural, and cosmetic industries as spices substances in foodstuffs, alcoholic beverages, and perfumes because of its spicy fragrance [20–22]. Oregano* (Origanum vulgare L.)* possesses an antibacterial property, and this is due mainly to its content of carvacrol and thymol and it possesses hydrophobicity characteristic, which results in the separation of the lipids from the bacterial membrane, disrupting the cell structure and making it much more permeable [23, 24]. The phenolic structure of thymol plays roles in antibacterial activity by entering the cell and permeabilizing the cytoplasm membrane, leading to a disturbed cellular metabolism [25].

The extensive investigation of the antimicrobial activities of honey and essential oils against a large category of bacterial and fungal pathogenesis is extended to test the combinations of both natural products [25, 26]. In all those studies it is found that there are positive interactions. Therefore, the main aim of the present work is to follow the changes in honey antibacterial activity, after the combination with* Origanum vulgare L.* essential oil, and figure out whether the honey physicochemical parameters influence the final activity.

## 2. Materials and Methods

### 2.1. Honey Samples

Twelve varieties of honey (H1 through H12) from different botanical origins were used in this study: Palestinian honeys (H1–H6) were purchased from a Palestinian beekeeper and Moroccan honeys (H7–H12) were purchased from a Moroccan beekeeper. All of the honey samples were collected in 2015 and stored at room temperature (22–24°C) in airtight plastic containers until analysis. The labels used in the study were taken from commercial containers and based on information from beekeepers who we bought those honeys samples.  Table 1 shows the labels of the used samples.

### 2.2. Essential Oils Extraction

The aerial parts (leaves and flowers) of the plant* Origanum vulgare L.* (EO) were bought from the herbalist in the region Imouzzer and were dried at room temperature, hydrodistilled for 4 h using a Clevenger type apparatus (Barnstead electrothermal, made in UK). The oils was dried over anhydrous sodium sulfate and stored in the dark at 2–4°C.

### 2.3. Free Acidity, pH, Ash, Electrical Conductivity, and Moisture

The measurements were carried out according to the Intl. Honey Commission IHC [27].

### 2.4. Honey Color and Melanoidins Estimations

Color was determined with a spectrophotometer by reading the absorbance in aqueous solutions at 635 nm (10 g honey in 20 mL water) [28]. Honey colors and their absorbance and mm pfund values were obtained using the following algorithm:

(1) $$\begin{array}{c} mm\;\;Pfund=-38.7+371.39\times Absorbance. \end{array}$$

Honey color was also determined spectrophotometrically by another method. The absorbance was measured at A560 and A720, and the net absorbance was calculated (A560–A720). Melanoidin content was estimated based on the browning index (net absorbance at A450–A720) [29]. The results were expressed as absorption units (AU).

### 2.5. Determination of Mineral Elements

The determination of potassium and sodium was performed by flame photometry using an air/butane flame. The determination of calcium and magnesium was performed by atomic absorption spectrometry [30].

### 2.6. Total Phenol Content

The total phenol content was determined according to the method described by Singleton and Rossi [31]. Total phenol content was expressed as the milligrams of the gallic acid equivalent per 100 gram of the honey mass (mg GAE/100 g).

### 2.7. Total Flavonoid Content

The total flavonoid content was determined according method described by Samatha et al. [32]. The flavonoid content was expressed as the milligrams of the quercetin equivalent per 100 gram of the honey mass (mg Q/100 g).

### 2.8. Total Flavonol Content

Total flavonol content was determined according method described by Engoor et al. [33]. The flavonol content was expressed as the milligrams of the quercetin equivalent per 100 gram of the honey mass (mg Q/100 g).

### 2.9. Estimation of Total Antioxidant Capacity by Phosphomolybdate Assay

The total antioxidant capacity was estimated by the phosphomolybdenum method according to the procedure described by Prieto et al. [34]. Total antioxidant capacity content was expressed as the milligrams of the ascorbic acid equivalent per 100 gram of the honey mass (mg AA/g).

### 2.10. DPPH Scavenging Assay

The DPPH radical scavenging activity assay was measured according to the method described by Brand-Williams et al. [35].

### 2.11. Determination of Reducing Power

The determination of reducing power assay was carried out as described by Oyaizu [36].

### 2.12. Bacterial Strains and Inoculums Standardization

In this study the antibacterial activity of EO, honeys, and mixtures (combination of honey and essential oil) were tested against sex bacterial strains: four Gram-negative strains* (Escherichia coli BLSE (ATB: 87) BGN, Escherichia coli (ATB: 57) B6N, Escherichia coli (ATB: 97) BGM, and Pseudomonas aeruginosa)* and two Gram-positive strains* (Streptococcus faecalis and Staphylococcus aureus)*.* Escherichia coli BLSE (ATB: 87) BGN, Escherichia coli (ATB: 57) B6N, and Escherichia coli (ATB: 97) BGM* were obtained from the Hassan II university Hospital and* Pseudomonas aeruginosa, Streptococcus faecalis, and Staphylococcus aureus* from the Laboratory of Microbiology, FMP, Fez were used as test microorganisms. Stock cultures were kept on Muller-Hinton agar under refrigeration (4°C). The inoculum suspension was obtained by taking colonies from 24-hour cultures. The colonies were suspended in sterile saline (0.9% NaCl) and shacked for 15 seconds. The density was adjusted to the turbidity of a 0.5 McFarland Standard (equivalent to 1–5 × 10^8^ cfu/mL) [37].

### 2.13. Agar Well Diffusion (AWD) Assay

The AWD assay was performed in triplicate based on the method of Kirby–Bauer [38]. With modification, Mueller Hinton agar plates are inoculated by swabbing from the standardized suspensions (10^8^ cfu/mL). Whatman paper discs (6 mm) are deposited on the surface of preinoculated agar. Next, the disks are impregnated with 5 *μ*L of EO. All plates were incubated at 37°C for 24 h. After incubation, the diameters of the inhibition zones were measured.

### 2.14. Determination of the Minimum Inhibitory Concentration (MIC)

The MICs were determined by microdilution assays in 96-well plates according to the standards of the NCCLS [39]. With modification, eight concentrations of EO and six concentrations of honey are prepared in sterile haemolysis tubes. They are carried out by successive dilutions 1/2 in distilled water ranging from 500 to 15.63 mg/ml for honeys and a mixture of Mueller Hinton (MH) and DMSO broth ranging from 10 to 0.09% for EO. The concentrations of honeys obtained in the well were between 250 and 7.81 mg/ml and between 1 and 0.009% for essential oil in such a way that the concentration of DMSO does not exceed 1% in the wells. Bacterial suspensions were prepared in the same way described previously. These suspensions were diluted in MH broth and plated in 96-well plates at a density of 5.0 × 10^5^ CFU well^−1^. Finally, different concentrations of honey and EO solutions were added to each well to determine the MIC values. After the plates were incubated at 37°C for 18, 40 *μ*l of 0.5% triphenyltetrazolium chloride (TTC) was added to each well. After 2 h of incubation, the MIC corresponds to the lowest concentration that does not produce red color [37].

### 2.15. Checkerboard Assay

The evaluation of interaction between honeys and EO was carried out according to the modified method of Nishio et al. [3]. Briefly, eight concentrations of EO and six concentrations of honeys were prepared in sterile tubes hemolyzed by dilutions 1/2. Then, honeys concentrations are introduced vertically into eight wells in a decreasing manner going from MIC × 2 to MIC/64, while the essential oil concentrations are introduced horizontally into seven wells in a decreasing fashion from CMI × 2 to CMI/16. The analysis of the combination was obtained by calculating the fraction inhibitory concentration index (FIC) using the following formula [26]:

(2) $$\begin{array}{c} \Sigma FICI=FIC\left(\;A\;\right)+FIC\left(\;B\;\right). \end{array}$$

FIC (*A*) = (MIC (*A*) in combination/MIC (*A*) alone); FIC (*B*) = (MIC (*B*) in combination/MIC (*B*) alone). The index values of the fractional inhibitory concentrations are interpreted as follows: FIC ≤ 0.5 = Synergy; FIC < 0.5–0.75 ≥= partial Synergy; FIC ≤ 0.76–1.0 >= Additive; FIC > 1–4 ≤= No interaction (not differential) and FIC > 4 = Antagonism.

### 2.16. Statistical Analysis

Statistical analysis was carried out by ANOVA through the GraphPad prism 6 program and using the Tukey post hoc test at *p* < 0.05. Correlations between the characterizing parameters of honey samples and between the honey physicochemical parameters and the FIC of honey and EOs combinations were achieved by Pearson correlation coefficient (*r*) at a significance level of 99% (*p* < 0.01). The results were also subjected to a multivariate analysis (principal component analysis). All experimental data were analyzed using MultiBiplot.

## 3. Results and Discussions

### 3.1. Preliminary Characterization

Visually, no honey sample showed signs of fermentation or granulation before physicochemical and antioxidant tests began. The results are summarized in Tables 1, 2, and 3.

Table 1 resumes the physicochemical characterization results, showing the conformity with the established standard [40], with some exceptions concerning the moisture content. The pH values of all analyzed samples ranged between 3.66 ± 0.01 and 4.48 ± 0.01; the free acidity values between 14.67 ± 2.08 and 36.33 ± 1.89 mEq/kg showed no sign of fermentation, as the normal value are below 50 mEq/kg [40]. The electrical conductivity correlated with the Ash content results (*R*^2^ = 0.7184; *p* < 0.01), and the minimum value was observed in sample H8 with an electrical conductivity value of 181.13 ± 0.25 *μ*S/cm and an Ash content value of 0.187 ± 0.003%, while sample H12 showed the highest value of both parameters 896.67 ± 8.50 *μ*S/cm and 0.507 ± 0.003% for electrical conductivity and Ash content, respectively. This sample exceeded the value of 800 *μ*S/cm established as the maximum value suitable for honey samples [41], which may be due to the richness of this honey in minerals. Electrical conductivity is considered as a very important parameter for determining the botanical origin of honey and thus differentiating between the honey of the nectar and that of the honeydew [42].

As mentioned before, samples H11 and H12 showed higher moisture content values (21 ± 0.2% and 21 ± 0.1%, resp.) than 20%, the maximum suitable for honey samples [27, 41]. Such increase may allow undesirable fermentation of honey by osmotolerant yeasts, which leads to the production of carbon dioxide and ethyl alcohol, which in turn oxidizes to acetic acid and water that are responsible for the bitter taste of honey [43]. Otherwise the remaining samples were within the norms, and a minimum value (15 ± 0.1%) was seen in sample H1.

Honey color is an indicator of the presence of compounds, such as phenols, terpenes, and carotenoids [28]. Such result was obtained in the analyzed samples of the present study, and the color correlated positively the phenols, flavonoids, and flavonol contents (*r* = 0.9378; *p* < 0.01, *r* = 0.9428; *p* < 0.01, *r* = 0.9336; *p* < 0.01, resp.). Honey varies between light white and amber colors and strongly correlated with melanoidin content (*r* = 0.9196; *p* < 0.01), confirming their participation in the resulting honey color [28, 29]. Detailed correlations between the analyzed parameters were illustrated in Table 7.

Table 2 shows the results obtained for mineral content of six Palestinian and six Moroccan honey samples. The potassium was the most abundant species in all the analyzed samples, with values ranging between 171.48 ± 0.50 in sample H6 and 2270.32 ± 0.29 mg/kg in sample H3. The sodium was the second most abundant, and varies between 59.79 ± 0.1 mg/kg and 285.67 ± 0.2 mg/kg, followed by calcium content 25.98 ± 0.22 mg/kg and 345.92 ± 1.07 mg/kg. The magnesium content was the less value amongst the analyzed minerals, which varies from a minimum value of 9.81 ± 0.39 mg/kg to a maximum of 77.53 ± 0.58 mg/kg. The richness of honey in minerals is a widely used parameter in determining the botanical and geographical origins of honey [44]. All values found in the samples were within the ranges reported for honeys by other studies [10, 45, 46].

### 3.2. Antioxidant and Antibacterial Activities

The determination of the antioxidant profile of the twelve honey samples was studied using six assays, and the results were summarized in Table 3, while the correlations with the phenolic, flavonoids, and flavonols contents were illustrated in Table 3. The total phenolic content values ranged from 12.91 ± 0.85 mg GAE/100 g in H8 to 89.53 ± 4.05 mg GAE/100 g in H9. For flavonoid content, the highest value was detected in H10 (50.41 ± 0.54 mg QE/100 g), and the minimum value was observed in sample H4 (2.86 ± 1.33 mg QE/100 g). Samples H4 and H10 conserved their order concerning the flavonol content and showed values of 1.34 ± 0.04 mg QE/100 g and 18.14 ± 0.90 mg QE/100 g, respectively. It has to be mentioned that sample H8, presenting the lowest content of phenolic composition, did not have a detectible level of flavonoids and flavonols, using the protocols described in Materials and Methods. Many authors have studied the content of phenolic compounds of honey and have suggested that its levels depend on the floral and geographical origins [47]. Correlations between phenols, melanoidins, and flavonoids may be attributed to the fact that all these compounds absorb light in the visible range, as previously reported by Aazza et al. [45]. In the present work phenolic content correlated positively with the flavonoids and flavonols contents (*r* = 0.9408; *p* < 0.0001 and *r* = 0.9538; *p* < 0.0001, respectively). The total antioxidant activity, expressed as mg of ascorbic acid equivalent/g honey (AAE) exceeded in samples H9 with a value of 181.43 ± 9.89 mg AAE/100 g and had the minimum value in sample H12 (133.76 ± 3.45 mg AAE/100 g). The sample H12 has also the best ability to scavenge the free radicals in the DPPH assay and to reduce the ferulic ions in the reducing power assay, with values IC_50_ = 5.61 ± 0.18 mg/mL and IC50 = 3.35 ± 0.04 mg/mL, respectively. The remaining samples had antioxidant activity that positively correlated with the phenolic content (*r* = −0.7570; *p* < 0.01 for DPPH activity, and *r* = −0.6014; *p* < 0.05).

The antibacterial activity of honeys was accomplished by the MICs assay. All honey samples revealed a positive result against the test pathogens.  Table 5 summarized the data of MICs of each honey sample against test bacterial strain. The MIC values of honeys ranged from 62.5 mg/ml to 250 mg/ml on six strains. Amongst the test bacterial strains,* Staphylococcus aureus* was the most sensitive and* Streptococcus faecalis* was most resistant. The results of MICs reported in this study are higher than those reported by Bouhlali et al. [48] and Mandal et al. [49], but similar to the results of MICs reported by Boukraa who found the MICs of honey ranging from 6% to 25% for* Staphylococcus aureus* and* Pseudomonas aeruginosa* [50]. The major antimicrobial properties of honey are known to be governed by to the hydrogen peroxide levels, as well as the nonperoxide factors that contribute to honey antibacterial and antioxidant activity (phenolic acids and flavonoids) [14, 15]. Studies have shown that the antibacterial activity varies according to the phytogeographical region and then the production of the different compositions [16, 51]; more recent studies reported the presence of other antimicrobial components, namely, antimicrobial peptide Bee defensin-1, HMF, and methylglyoxal (MGO), but also phenolic compounds such as flavonoids [3].

In our study, the used essential oil of* Origanum vulgare L.* in the agar diffusion assay against the Gram-negative and Gram-positive bacterial strains studied gave an diameter of inhibition of 51.25 ± 1.41 mm with* Escherichia coli* of serotype 87, A diameter of 46 ± 1.41 mm with* Staphylococcus aureus*, a diameter of 40.25 ± 6.01 mm with* Streptococcus faecalis*, a diameter of 33.5 ± 3.54 mm with* Escherichia coli* serotype 97, and a diameter of 32.5 ± 3.54 mm with* Escherichia coli* serotype 57 and finally the most small diameter of inhibition was that with* Pseudomonas aeruginosa *13.25 ± 1.77 mm. The determination of the MICs of essential oil on different bacteria strains showed that Gram-negative strains are more sensitive than Gram-positive bacteria (Table 4).

### 3.3. Antimicrobial Activity of Combination of Honey Samples and* Origanum vulgare L*. Essential Oil

The test used was the checkerboard assay in order to measure the inhibitory activity of the mixture by determining the fractional inhibitory concentration (FIC).  Table 6 shows the twelve honey samples tested against six strains in combination with the essential oil. The Fractional inhibitory concentration (FIC) was used to evaluate the synergistic activity. From 72 combinations, 3 (4.16%) had total synergism, 27 (37.5%) showed a partial synergistic and additive interaction, 38 (52.77%) had no interaction, and 4 (4.44%) had no effect on bacteria strains. The best synergistic effect was obtained with the combination of essential oil and H11 honey sample; this effect was observed on two bacteria (*Escherichia coli (ATB: 97)* and* Streptococcus faecalis*) with FIC values being 0.312 and 0.156, respectively, and the combination of essential oil and H1 honey sample on* Streptococcus faecalis* with FIC values was 0.31.

Combining honey with essential oil reduces the MICs of the honeys 1–4-fold and 1–8-fold for Gram-positive and Gram-negative bacteria, respectively. The weakest synergism was obtained with essential oil-honey against* Escherichia coli (ATB: 57)* with FIC ranging from 0.75 to 2.25. Several studies have shown the interaction between honey and other substances [25, 50, 52]; in all of these studies it is found that there are positive interactions using the isobolograms method, but these can be considered additives according to fractional inhibitory concentration index (FIC) [53]. This synergistic interaction appeared to be due to different mechanisms which included inhibition of protective enzymes and sequential inhibition of common biochemical pathways [54].

### 3.4. Correlations and Multivariate Analysis

**Figure 1:**
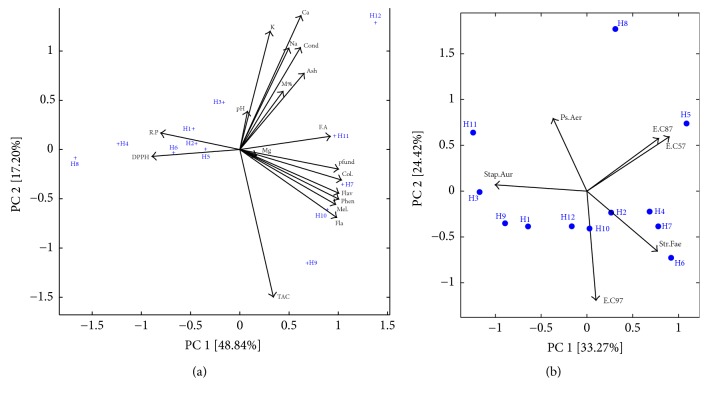
Principal component analysis (PCA). (a) Biplots of the analyzed honey samples using the physicochemical parameters as an input. K: potassium; Na: sodium; Ca: calcium; Mg: magnesium; pH; DPPH; M: moisture; Cond: conductivity; Ash; pfund; Col: color; Fla: flavonoid; Fla: flavonol; Phen: phenol; TAC; Mel: melanoidin. (b) PCA biplot of the analyzed honey samples using the resulting FIC of combining honeys with EO as an input. E. C87:* Escherichia coli BLSE (ATB: 87) BGN*; E. C57:* Escherichia coli (ATB: 57) B6N*; E. C97:* Escherichia coli (ATB: 97) BGM*; Stap. Aur:* Staphylococcus aureus*; Ps. Aes:* Pseudomonas aeruginosa*; Str. Fae:* Streptococcus faecalis*.

Honey samples distribution and homogeneity based on their physicochemical parameters were studied using principal component analysis, as a powerful tool for the chemometric analysis [55]. The results were illustrated in Figure 1(a). The first component explained 48.84% and represented in its positive part color, phenolic compounds, and flavonoids, while DPPH IC50 and the reducing power were the dominating parameters in the negative part. The second principal component explained 17.20% of the given data and represented mainly the TAC activity in the negative part and the conductivity, Ash content, and mineral composition in the positive part.

Based on the used parameters, a good geographical discrimination was made between the Palestinians and Moroccan honey samples, which were discriminated by the first component. H8 originating from Morocco was the only exception and was misloaded with the Palestinian samples. Moroccan honey samples were in the right part of the plot and characterized by their homogeneity in terms of polyphenol, flavonoids, and flavonols, which implicated the positive correlation with colors, melanoidins estimations, and negatively the IC50 of DPPH activity. Those samples had also the highest value of the minerals contents, which was also reflected by the Ash content and electrical conductivity. Palestinians samples were in the opposite side of the plot and correlated with the reducing power.

The use of the resulting FIC values after combining both honey samples and the EO to run the PCA showed little effect of the geographical origin (Figure 1(b)). Each of the two main clusters was a mixture of samples belonging to both geographical origins and was discriminated by the first principal component, which conserved 33.27% of the given data. The first cluster (the orange shadow area) was formed by honey samples H1 and H2 from Palestine, and samples H9 and H12 from Morocco. The cluster was loaded in the negative part of the first principal component linking the gained FIC value against* Staphylococcus aureus* after the combination. The second cluster was in the right part of the plot linking the FICI values against* Streptococcus faecalis.*

Despite their botanic origin, samples originating from Morocco presented high amounts of mineral content, and so, high Ash content and electrical conductivity, except for sample H8. The same phenomenon was observed in their phenols, flavonoids, and flavonols contents, which also explained their high antioxidant activity. The observation made about the minerals content can be explained by their different geographical origins, as it is well known in the literature [44]. Bioactive compounds content or the antioxidant activities are more likely to be influenced by the botanic origin of honey samples [56].

Considering the investigation of the effect of physicochemical parameters of honey sample on the final activity, after combination with the EO, correlation study between the assessed parameters and the FIC of the combination of each honey sample was made, and the results were illustrated in Table 8. The melanoidins content was showed to be positively influencing the gained antibacterial activity after the combination with EO, and its values presented negative correlation with the FIC values against* Escherichia coli* (ATB: 57), and* Streptococcus faecalis* (*p* < 0.05). The resulting FIC against the same bacterial strains was positively influenced by the phenolic content, but a significant negative correlation was only seen in the case* Escherichia coli* (ATB: 57). Negative correlations were also seen between the FIC against* Escherichia coli* (ATB: 57) from one side, and the electrical conductivity (*p* < 0.05) and the mineral content (*p* < 0.01) from the other side. The free acidity had a significant (*p* < 0.05) positive effect on the gained FICI against* Escherichia coli (ATB: 57).*

The reported correlations may indicate a specific pathway of honey effect on the bacterial strains. Previous studies reported the importance of melanoidins and phenolic contents on honey antimicrobial activity and include them in the nonperoxide way [14, 15].

To investigate the effect of the initial activity against a bacterial strain on the gained interaction after the combination with the EO, correlation study was accomplished, and the results are illustrated in Table 9. In general, no significant influence was recorded. The initial honey activity against* Escherichia coli* (ATB: 97) seems to have a significant influence on the resulting activity after the combination with EO. A significant (*p* < 0.05) negative correlation was obtained between the MICs of honey samples against* Escherichia coli* (ATB: 97), and the FIC of the combination against the same bacterial strain.

## 4. Conclusion

Boosting the antimicrobial effect of honey using essential oils seems to be a promising way to investigate new pathways in the development of new antimicrobial drugs. The present work showed a synergetic effect after mixing both natural products and revealed a possible influence of honey physicochemical properties, namely, the melanoidins, phenolic contents, and the free acidity.

The observed interactions are influenced by the botanic origin of honey, and the used bacterial strains, which suggest the necessity of extending the research to investigate more honey samples and bacterial strains. In addition maximum characterizing parameters need to be probed to allow a clearer image about the possible influencing factors.

## Figures and Tables

**Table 1:** Honey samples purchase information and physicochemical characterization.

Sample code	Local appellation	Scientific name of the plant	Region name	pH	Free acidity (mEq/kg)	Electrical conductivity (*μ*S/cm)	Moisture (%)	Ash (%)	Color (A560–A720)	Melanoidin (A450–A720)	Pfund scale (mm)	Honey color
H1	Morar	*Centaurea dumulosa*	Al-Khalīl	4.32 ± 0.01^i^	21.00 ± 1.00^ab^	573.67 ± 5.03^c^	15 ± 0.1^f^	0.320 ± 0.003^b^	0.14 ± 0.00^cd^	0.49 ± 0.01^f^	42.94 ± 1.22^e^	Light extra amber
H2	Morar akhdar	*Echinops spinosissimus*	Al-Khalīl	3.71 ± 0.01^b^	30.17 ± 2.08^abc^	415.00 ± 5.57^f^	17 ± 0.1^bcd^	0.080 ± 0.00^j^	0.14 ± 0.00^cd^	0.93 ± 0.02^d^	35.08 ± 2.29^ef^	Light extra amber
H3	Zohif	*Thymus capitatus*	Al-Khalīl	4.09 ± 0.01^g^	27.00 ± 1.32^abc^	554.00 ± 2.65^cd^	16.75 ± 0.00^bcde^	0.109 ± 0.006^h^	0.19 ± 0.01^c^	0.65 ± 0.02^e^	50.97 ± 0.86^e^	Light amber
H4	Rabat	*Conyza bonariensis*	Ramallah	3.66 ± 0.01^a^	23.67 ± 1.89^ab^	273.00 ± 4.58^h^	17.9 ± 0.05^b^	0.093 ± 0.001^i^	0.09 ± 0.01^cd^	0.28 ± 0.01^g^	10.53 ± 199^g^	Extra white
H5	Multiforal	*Thymus capitatus* *Origanum syriacum*	Ramallah	3.80 ± 0.02^c^	30.25 ± 3.73^abc^	368.00 ± 1.73^g^	17.3 ± 0.2^bcd^	0.178 ± 0.006^g^	0.20 ± 0.04^c^	0.64 ± 0.05^e^	51.73 ± 5.63^e^	Light amber
H6	Sader	*Ziziphus spina-christi*	Jericho	4.48 ± 0.01^j^	16.67 ± 1.04^a^	688.67 ± 3.06^b^	17.6 ± 0.2^bc^	0.222 ± 0.00^f^	0.15 ± 0.01^c^	0.55 ± 0.00^f^	42.05 ± 1.10^e^	Light extra amber
H7	Zaitra	*Thymus vulgaris L.*	Timahdite	3.90 ± 0.01^e^	37.83 ± 2.25^abcd^	449.00 ± 6.00^e^	16.4 ± 0.1^bcde^	0.235 ± 0.001^e^	0.33 ± 0.01^ab^	1.10 ± 0.02^c^	132.07 ± 3.03^b^	Dark amber
H8	Limon	*Citrus limon L*	Khnichet	4.03 ± 0.01^f^	14.67 ± 2.08^a^	181.13 ± 0.25^i^	18.2 ± 0.1^b^	0.183 ± 0.006^g^	0.07 ± 0.01^cdf^	0.21 ± 0.02^h^	4.07 ± 3.38^g^	Water white
H9	Bochnikha	*Ammi visnaga L.*	Had Kourt	4.03 ± 0.01^f^	30.08 ± 2.01^abc^	425.67 ± 2.08^f^	17.9 ± 0.00^b^	0.187 ± 0.003^g^	0.32 ± 0.01^ab^	1.43 ± 0.02^a^	92.18 ± 1.79^d^	Amber
H10	Kabar	*Capparis spinosa*	Moulay Yacoub	4.01 ± 0.01^f^	29.33 ± 1.76^abc^	422.33 ± 2.52^f^	17.9 ± 0.2^b^	0.272 ± 0.00^d^	0.35 ± 0.01^a^	1.49 ± 0.02^a^	126.09 ± 3.68^b^	Dark amber
H11	Daghmos	*Euphorbia L.*	Lakhsas	3.85 ± 0.01^d^	34.17 ± 0.76^abcd^	562.00 ± 2.65^c^	21 ± 0.2^a^	0.309 ± 0.001^c^	0.38 ± 0.01^a^	1.26 ± 0.02^b^	161.99 ± 6.34^a^	Dark amber
H12	Arbousie	*Arbutus unedo*	Chefchaouen	4.25 ± 0.01^h^	36.33 ± 1.89^abcd^	896.67 ± 8.50^a^	21 ± 0.1^a^	0.507 ± 0.003^a^	0.32 ± 0.02^ab^	1.16 ± 0.01^c^	109.15 ± 2.14^c^	Amber

Values in the same column followed by the same letter are not significantly different (*p* < 0.05) by Tukey's multiple range test.

**Table 2:** Mineral content of honey samples.

Sample code	Potassium (mg/kg)	Sodium (mg/kg)	Calcium (mg/kg)	Magnesium (mg/kg)	Na + K +Ca + Mg (mg/kg)
H1	926.01 ± 0.90^d^	106.12 ± 0.9^k^	154.55 ± 5.28^f^	27.73 ± 0.32^e^	1214.41
H2	955.88 ± 1.14^c^	159.28 ± 0.29^g^	145.77 ± 1.13^g^	15.42 ± 0.42^gh^	1276.35
H3	2270.32 ± 0.29^a^	213.55 ± 0.40^c^	173.84 ± 1.95^d^	42.54 ± 0.55^c^	2700.25
H4	367.12 ± 0.96^i^	156.43 ± 0.45^h^	126.42 ± 1.07^hi^	14.44 ± 1.23^gh^	663.9
H5	576.86 ± 0.43^g^	176.7 ± 0.26^f^	133.46 ± 3.01^h^	24.50 ± 0.20^f^	911.52
H6	171.48 ± 0.50^k^	59.79 ± 0.1^m^	129.49 ± 1.1^h^	9.81 ± 0.39	370.57
H7	830.26 ± 1.52^e^	256.64 ± 0.28^b^	166.19 ± 0.85^e^	62.17 ± 0.92^b^	1315.26
H8	424.24 ± 0.41^h^	114.74 ± 0.44^i^	79.35 ± 4.04^j^	25.76 ± 0.56^f^	644.55
H9	286.78 ± 1.21^j^	111.16 ± 1.27^j^	25.98 ± 0.22^k^	17.44 ± 0.05^g^	458.8
H10	598.43 ± 0.39^f^	75.39 ± 0.20^l^	188.50 ± 1.32^c^	15.69 ± 0.46^g^	878.01
H11	579.75 ± 2.17^g^	179.77 ± 0.06^d^	262.57 ± 1.73^b^	35.16 ± 0.84^d^	1054.75
H12	1723.20 ± 1.21^b^	285.67 ± 0.20^a^	345.92 ± 1.07^a^	77.53 ± 0.58^a^	2432.32

Values in the same column followed by the same letter are not significantly different (*p* < 0.05) by Tukey's multiple range test.

**Table 3:** Bioactive compounds estimations and antioxidant activities of honey samples.

Sample code	Phenols (mg GAE/100 g)	Flavonoids (mg QE/100 g)	Flavonol (mg QE/100 g)	TAC (mg AA/g)	DPPH (IC50 = mg/mL)	Reducing power (IC50 = mg/mL)
H1	32.49 ± 0.08^de^	13.43 ± 0.16^e^	6.07 ± 0.04^de^	145.13 ± 8.85^a^	20.88 ± 0.36^d^	4.18 ± 0.70^ab^
H2	33.20 ± 0.29^de^	9.39 ± 0.02^efg^	8.86 ± 0.14^d^	134.46 ± 5.94^ab^	31.74 ± 0.31^ef^	3.15 ± 0.64^a^
H3	42.13 ± 2.17^d^	12.72 ± 0.28^ef^	7.93 ± 0.49^d^	142.87 ± 7.15^a^	26.36 ± 0.25^e^	3.05 ± 0.08^a^
H4	17.97 ± 0.98^f^	2.86 ± 1.33^i^	1.34 ± 0.04^e^	134.61 ± 9.12^ab^	89.49 ± 1.03^g^	5.55 ± 0.36^abc^
H5	42.66 ± 2.24^d^	17.19 ± 1.36^e^	7.69 ± 0.22^d^	140.32 ± 4.62^a^	26.09 ± 0.58^e^	6.06 ± 0.33^abc^
H6	37.50 ± 2.07^d^	7.47 ± 0.35^efg^	6.67 ± 0.04^de^	153.94 ± 5.62^a^	38.09 ± 0.26^f^	6.60 ± 0.25^abc^
H7	74.05 ± 1.21^b^	59.33 ± 1.07^a^	15.08 ± 0.04^b^	163.51 ± 8.55^a^	10.85 ± 0.02^b^	1.60 ± 0.97^a^
H8	12.91 ± 0.85^f^	ND	ND	148.13 ± 4.17^a^	91.46 ± 1.91^g^	13.06 ± 0.65^d^
H9	89.53 ± 4.05^a^	46.52 ± 0.56^b^	18.14 ± 0.90^a^	181.43 ± 9.89^a^	16.84 ± 0.68^c^	1.82 ± 0.19^a^
H10	86.66 ± 1.31^a^	50.41 ± 0.54^b^	17.94 ± 0.20^a^	158.90 ± 4.99^a^	21.23 ± 0.18^d^	3.54 ± 1.05^a^
H11	64.54 ± 2.13^c^	42.30 ± 0.07^bc^	15.50 ± 0.56^b^	143.83 ± 2.30^a^	29.36 ± 0.96^ef^	3.81 ± 0.02^ab^
H12	78.45 ± 1.24^b^	45.70 ± 2.29^bc^	12.54 ± 0.33^c^	133.76 ± 3.45^ab^	5.61 ± 0.18^a^	3.35 ± 0.04^a^

Values in the same column followed by the same letter are not significantly different (*p* < 0.05) by Tukey's multiple range test; ND: not determined.

**Table 4:** Diameters of the inhibition zones (mm) generated by EOof *Origanum vulgare L.* against different bacterial strains.

Sample code		*E. coli BLSE (ATB: 87) BGN*	*E. coli (ATB: 57) B6N*	*E. coli (ATB: 97) BGM*	*Pseudomonas aeruginosa*	*Streptococcus faecalis*	*Staphylococcus aureus*
EO	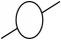	51.25 ± 1.41	32.5 ± 3.54	33.5 ± 3.54	13.25 ± 1.77	40.25 ± 6.01	46 ± 1.41
	MIC (%)	0.125	0.125	0.5	0.5	1	1

EO: essential oil, *n* = 3.

**Table 5:** MICs values of honey samples used individually or in combination against Gram-positive and Gram-negative bacteria.

Sample code	*Escherichia coli BLSE (ATB: 87) BGN*		*Escherichia coli (ATB: 57) B6N*		*Escherichia coli (ATB: 97) BGM*		*Pseudomonas aeruginosa*		*Streptococcus faecalis*		*Staphylococcus aureus*	
	MIC alone	MIC combined	MIC alone	MIC combined	MIC alone	MIC combined	MIC alone	MIC combined	MIC alone	MIC combined	MIC alone	MIC combined
H1	125	62.5	250	125	250	125	125	62.5	250	62.5	125	125
H2	62.5	62.5	125	125	250	125	125	62.5	250	125	62.5	31.25
H3	125	62.5	250	125	250	125	125	125	250	125	62.5	125
H4	125	125	125	250	250	250	125	125	250	250	125	125
H5	250	250	125	250	-	-	250	250	250	250	-	-
H6	125	125	125	250	250	250	250	125	250	250	125	125
H7	125	250	125	125	250	250	125	62.5	250	125	62.5	31.25
H8	125	250	125	250	-	-	250	250	-	-	250	250
H9	125	62.5	125	125	250	250	125	125	250	125	62.5	125
H10	125	62.5	125	125	250	125	125	62.5	250	125	62.5	31.25
H11	125	62.5	125	125	250	62.5	125	125	250	31.25	62.5	125
H12	250	250	250	125	250	250	250	250	250	125	250	125

H1–H12 honeys; MIC was defined as 99% bacteriostatic effects; *n* = 3, - = Not effective.

**Table 6:** Honey-EO interaction evaluation using the FIC.

Sample code	*Escherichia coli BLSE (ATB: 87) BGN*		*Escherichia coli (ATB: 57) B6N*		*Escherichia coli (ATB: 97) BGM*		*Pseudomonas aeruginosa*		*Streptococcus faecalis*		*Staphylococcus aureus*	
	FIC	Interaction	FIC	Interaction	FIC	Interaction	FIC	Interaction	FIC	Interaction	FIC	Interaction
H1	1	A	1	A	0.75	PS	0.625	PS	0.3125	S	1.125	NI
H2	1.5	NI	1.5	NI	0.75	PS	1	A	0.5625	PS	0.625	PS
H3	1	A	1	A	0.625	PS	1.25	NI	0.5625	PS	2.0625	NI
H4	1.5	NI	2.5	NI	1.5	NI	1.25	NI	1.03125	NI	1.0625	NI
H5	1.5	NI	2.25	NI	-	-	1.5	NI	1.0625	NI	-	-
H6	1.5	NI	2.5	NI	1.125	NI	0.75	PS	1.03125	NI	1.125	NI
H7	2.5	NI	1.25	NI	1.25	NI	0.625	PS	0.53125	PS	0.531	PS
H8	2.5	NI	3	NI	-	-	1.25	NI	-	-	1.125	NI
H9	1	A	1.5	NI	1.5	NI	1.25	NI	0.5625	PS	2.125	NI
H10	1	A	1.5	NI	0.625	PS	0.625	PS	0.5625	PS	0.5625	PS
H11	1	A	1.5	NI	0.312	S	1.25	NI	0.15625	S	2.0625	NI
H12	1.5	NI	0.75	PS	1.125	NI	1.25	NI	0.53125	PS	0.5625	PS

FIC was the fractional inhibitory concentration; S = synergy, PS = partial synergy, A = additive, NI = no Interaction, and - = not effective.

**Table 7:** Pearson correlation coefficients between the assessed physicochemical parameters of honey samples.

	Phenols	Flavonoids	Flavonol	TAC	DPPH	Reducing power	Color	Melanoidin	Electrical conductivity	Ash
Phenols	1	0.9408^*∗∗∗∗*^	0.9538^*∗∗∗∗*^	0.5915^*∗*^	−0.7570^*∗∗*^	−0.6014^*∗*^	0.9378^*∗∗∗∗*^	0.9444^*∗∗∗∗*^	-	-
Flavonoids	0.9408^*∗∗∗∗*^	1	0.9202^*∗∗∗∗*^	-	−0.6400^*∗*^	-	0.9428^*∗∗∗∗*^	0.8714^*∗∗∗*^	-	-
Flavonol	0.9538^*∗∗∗∗*^	0.9202^*∗∗∗∗*^	1	0.6682^*∗*^	−0.6138^*∗*^	-	0.9336^*∗∗∗∗*^	0.9780^*∗∗∗∗*^	-	-
TAC	0.5915^*∗*^	-	0.6682^*∗*^	1	-	-	-	-	-	-
Color	0.9378^*∗∗∗∗*^	0.9428^*∗∗∗∗*^	0.9336^*∗∗∗∗*^	-	−0.7105^*∗∗*^	-	1	0.9196^*∗∗∗∗*^	-	-
Melanoidin	0.9444^*∗∗∗∗*^	0.8714^*∗∗∗*^	0.9780^*∗∗∗∗*^	-	−0.7174^*∗∗*^	−0.6169^*∗*^	0.9196^*∗∗∗∗*^	1	-	-
Electrical conductivity	-	-	-	-	−0.6627^*∗*^	-	-	-	1	0.7184^*∗∗*^
Ash	-	-	-	-	-	-	-	-	0.7184^*∗∗*^	1

^*∗*^Correlation is significant at the *p* < 0.05 level. ^*∗∗*^ Correlation is significant at the *p* < 0.01 level. ^*∗∗∗*^Correlation is significant at the *p* < 0.005 level. ^*∗∗∗∗*^Correlation is significant at the *p* < 0.0001 level, -: Not significant.

**Table 8:** Pearson correlation coefficients between the honey physicochemical parameters and the FIC of the combinations.

	FIC *Escherichia coli BLSE (ATB: 87) BGN*	FIC *Escherichia coli (ATB: 57)*	FIC *Escherichia coli (ATB: 97)*	FIC *Pseudomonas aeruginosa*	FIC *Staphylococcus aureus*	FIC *Streptococcus faecalis*
Free acidity	-	−0.641^*∗*^	-	-	-	-
Phenols	-	−0.593^*∗*^	-	-	-	-
Melanoidin	-	−0.584^*∗*^	-	-	-	−0.607^*∗*^
Mineral content	-	−0.711^*∗∗*^	-	-	-	-

^*∗*^Correlation is significant at the *p* < 0.05 level. ^*∗∗*^Correlation is significant at the *p* < 0.01 level. Correlation is significant at the *p* < 0.005 level. Correlation is significant at the *p* < 0.0001 level, -: not significant.

**Table 9:** Pearson correlation coefficients between MICs of honey samples and the FIC of the combinations.

	FIC *Escherichia coli BLSE (ATB: 87) BGN*	FIC *Escherichia coli (ATB: 57)*	FIC *Escherichia coli (ATB: 97)*	FIC *Pseudomonas aeruginosa*	FIC *Staphylococcus aureus*	FIC *Streptococcus faecalis*
MICs of honeys on *Escherichia coli BLSE (ATB: 87) BGN*	0.0242					
MICs of honeys on *Escherichia coli (ATB: 57)*		−0.6564^*∗*^				
MICs of honeys on *Escherichia coli (ATB: 97)*			0.04684			
MICs of honeys on *Pseudomonas aeruginosa*				0.3197		
MICs of honeys on *Staphylococcus aureus*					0.5543	
MICs of honeys on *Streptococcus faecalis*						−0.02040

^*∗*^Correlation is significant at the *p* < 0.05 level. Correlation is significant at the *p* < 0.01 level. Correlation is significant at the *p* < 0.005 level. Correlation is significant at the *p* < 0.0001 level, Not significant.
